# Zoogeochemists Measure How Muddy Animals Change Their Environments’ Chemistry

**DOI:** 10.1021/acscentsci.4c00159

**Published:** 2024-02-08

**Authors:** Robin Donovan

Chris Dutton is used to broken equipment. In the years he’s spent analyzing the creatures of the Mara River basin in Africa, his cameras and sensors have been tusked by elephants, crushed by hippos, and mauled by predatory cats. After his tech disappears, it rarely returns. So when his phone buzzed with a message that a single wildlife camera had been recovered by a local road worker more than a kilometer from his research site in southern Kenya’s Narok County, the ecologist was intrigued.

**Figure:**
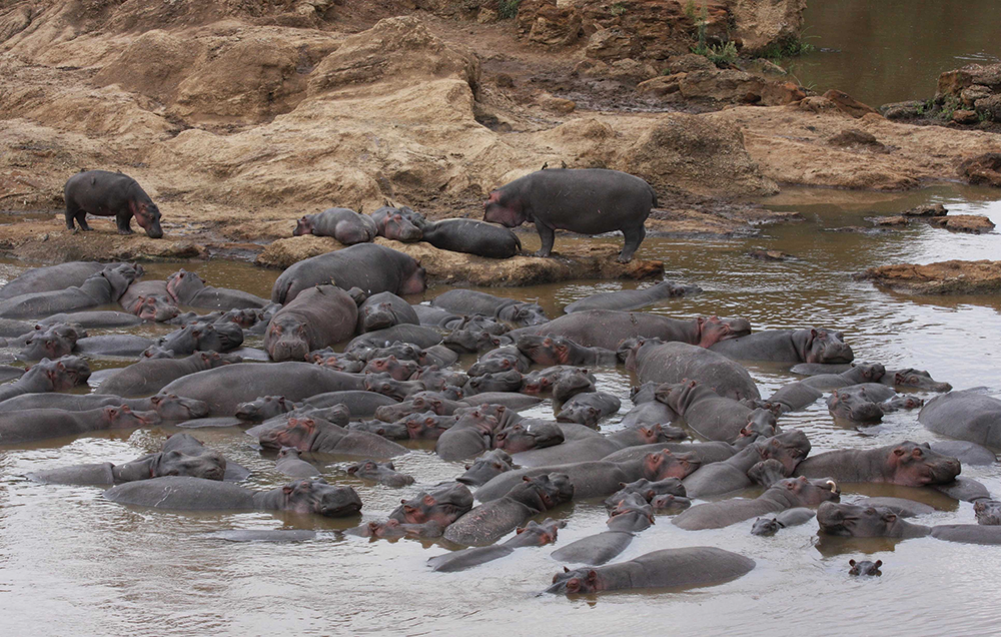
A throng of hippos in the Mara River means a spike in waste deposits and, possibly, carbon dioxide and methane emissions. Credit: Chris Dutton.

“I can’t even remember how many thousands and thousands of dollars of equipment we’ve lost in the Mara River,” he says. After retrieving the camera, he spotted the perpetrator on the recorded video a hyena peeking curiously at the lens before grabbing the shiny treasure in its mouth and sprinting across the savannah.

The camera is just one tool in a yearslong study of how animals in the Mara River basin are active participants in the chemistry of their environment. Dutton, an ecologist at the University of Florida, has taken an interest in the hippopotamus, whose numbers and nightly saunters are captured on camera.

Over the course of the dry season, the waterway where hippos thermoregulate can shift from an idyllic, flowing river into stagnant, stinky pools. These enormous mammals may just be cooling off, but in doing so, they might also be altering the whole region’s nutrient cycle, Dutton believes.

He’s checking to see if the gut bacteria from hippo droppings are flourishing after they’ve been jettisoned into the pools. If he’s right, something as virtuous as hippo conservation may be chemically shifting the ponds as low oxygen levels let these anaerobic bacteria begin their own breakdowns, ultimately releasing carbon dioxide and methane gases that float away into the atmosphere.

Hippos are among a myriad of creatures whose everyday behaviors shape and shift ecosystem conditions. Foraging and trampling by grazers, mass births and deaths of migratory herds, and even hippos taking a quick dip can have sizable ripple effects on carbon and nitrogen cycling in these ecosystems. Animal behaviors can’t be classified as good or bad—and most ecologists don’t see them that way. But as climate change accelerates, ecosystem conditions are becoming less stable and less predictable, potentially amplifying the ripple effect of animal behaviors.

## Fluxers and lizard squeezers unite

Dutton is part of a nascent band of zoogeochemists, ecologists who study how animals physically move nutrients around their ecosystems. The field gained momentum in 2018 when a group of Yale University ecologists published the results of a study outlining the impact of animals on carbon cycling. We know that animals “fundamentally change the properties of the ecosystem when they’re present,” says Oswald Schmitz, one of the coauthors of the article.

Zoogeochemists bridge two factions in their field. Peter Groffman, an ecosystem ecologist at the City University of New York, describes the divide as “lizard squeezers,” or population ecologists, versus “fluxers.” The latter group studies changes in ecosystems’ carbon and nitrogen cycles—the dynamics that govern where these elements go in an environment and thus how much carbon is stored or released by plants and animals and how much life-sustaining nitrogen is available to them over time. These cycles are essential to the structure and nourishment, respectively, of all living things.

In past decades, Groffman says, “you had a bunch of population people who were very grouchy about the ecosystem stuff.” Some ecologists assumed that animals simply weren’t abundant enough to have an appreciable impact, and there wasn’t a great way to prove them wrong.

But more sophisticated and economical remote sensing equipment—like Dutton’s abused cameras—makes those impacts easier to quantify. For example, hand-held devices with X-ray fluorescence capabilities can now be used for elemental analyses in the field, and affordable GPS monitors help researchers track animal movements. Machine learning and artificial intelligence can automatically identify animals in wildlife camera footage and process massive data sets to follow nutrient transport among species.

These tools allow scientists to track elements in the environment with individual-level specificity—and filter out growing human influences. The more extreme the environment, the bigger the impact individuals have. “These animal effects are hidden in plain sight, and we’re ignoring them,” Schmitz says.

## Cool dips, dramatic consequences

University of Florida ecologist Amanda Subalusky says some ecosystem ecologists still believe that the animal impacts she studies are outliers and don’t have the ecological influence of, say, plants. Subalusky’s research on wildebeest die-offs has enlivened the debate, adding evidence that animals regularly trigger substantial shifts in carbon and nutrient cycling in their ecosystems.

In one study, Subalusky measured how mass wildebeest drownings change nutrient cycling in the Mara River. The meat that the Nile crocodiles and birds pick off the wildebeests’ bones provides the ecosystem with a quick hit of nutrients, and her research showed that the bones themselves support life in the river by releasing carbon, nitrogen, and phosphorus onto the riverbed.

Dead animals can provide a smorgasbord of concentrated nutrients wherever they fall. Although dead plants far outnumber dead animals, carcasses break down faster, rapidly infusing the soil with nutrients, which soon become available to plants and other organisms.

Protein-rich muscle tissue breaks down into amino acids, which plants quickly absorb. Fatty tissue is loaded with labile carbon, a form that’s more accessible to microbes than carbon from, say, decomposing plants. And those fueled-up microbes in turn speed the decomposition of nearby plant matter.

In contrast, it takes microbes months to break down cellulose-rich plant material, so nutrients from fallen leaves might not be available until the following summer, Schmitz says. The effect “might be localized, but animals introduce huge amounts of nutrients that boost productivity much faster than microbes that release soil nutrients for plants.”

Animals also forage. They speedily redirect nutrients by nibbling on leaves—sometimes naturally picking those richest in nitrogen—and then urinating far from where they ate, for example. Without that consumption and transport, those nutrients might otherwise linger in the soil in a less-digestible form of decaying plant material. Such behaviors turn animals into “ecological engineers,” Groffman says.

Subalusky and Dutton are now studying the chemical shifts orchestrated by Kenya’s hippos, which depend on ponds for temperature regulation. Fed by the Mara River, the ponds form as portions of the river disappear in the dry season, leaving a series of isolated pools “like pearls on a string,” Subalusky says.

**Figure:**
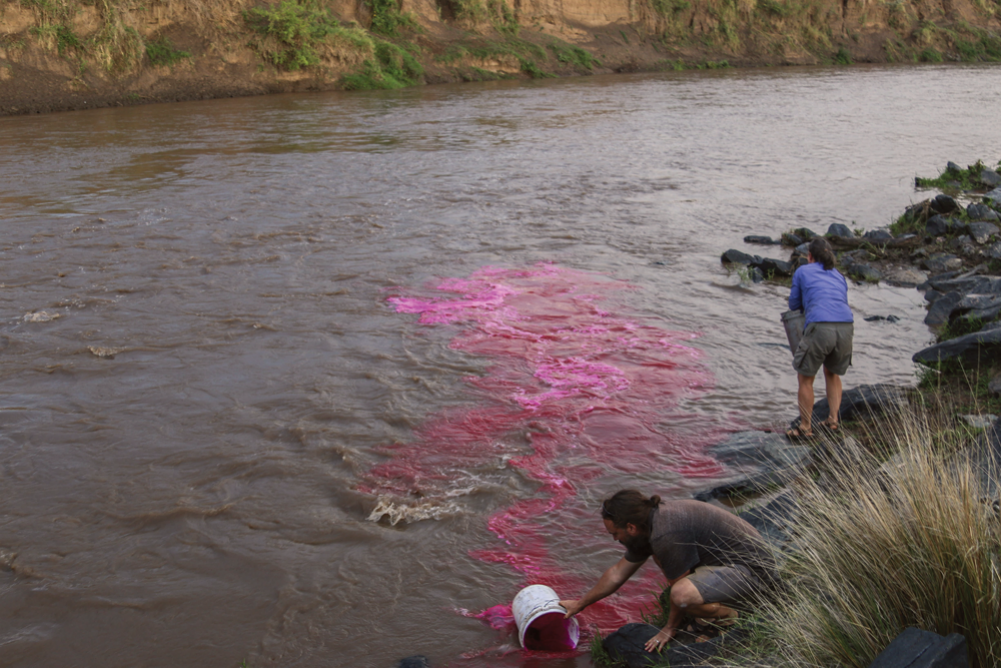
Researchers Amanda Subalusky (right) and Chris Dutton use rhodamine, an inert chemical tracer, to track nutrient uptake. Credit: Chris Dutton.

Groups of hippos that nap in, graze near, and wade through the ponds also defecate and urinate, transporting carbon and nutrients from land to water and back. But as climate change boosts average temperatures, river levels drop more readily, and the water flow that normally flushes the ponds arrives less frequently. So the team thinks that, in an indirect way, hotter weather could cause the buildup of hippo waste—and thus hippo gut microbes—in the ponds.

Subalusky and Dutton are now testing a hypothesis that the vast stable of microbes in hippo stool spurs carbon and nitrogen cycling in these pond ecosystems. They’ve also found that methane and carbon dioxide emissions from poop-laden ponds often increase as flushing wanes. Underwater, the bacteria may mediate and supercharge chemical processes, including the consumption of oxygen and the purging of carbon from the ecosystem as carbon dioxide and methane are generated and bubble off into the atmosphere.

Using sensors and water samples from 20 artificial streams, Dutton is comparing carbon, nitrogen, dissolved oxygen, and pH levels in waterways with typical, bacteria-laden feces versus sterilized excrement to isolate the microbiome’s effects. Early measurements show that gut bacteria-laden ponds have increased carbon emissions and lower dissolved-oxygen concentrations.

These seemingly small changes in a pond’s chemistry can have ripple effects in the environment. As oxygen levels plummet, some anaerobic microbes use nitrate instead of oxygen for respiration. In that process, nitrate is eventually reduced to nitrous oxide, a gas that floats off and takes some of the ecosystem’s nitrogen with it. Similarly, anoxic conditions lead to the release of phosphorus from sediment, which can provoke algal or cyanobacterial blooms, which can both be toxic. And when organic material decomposes, emissions of carbon dioxide and methane rise and pH drops. Each shift is a potential threat to the species that thrive under certain conditions and depend on the pond for food, water, or shelter.

Ponds with higher water levels benefit from frequent flushing, which delivers oxygenated water and prevents nutrient buildup. Fewer nutrients mean less chance of overgrowth of bacteria that consume dissolved oxygen and lead to anoxia.

Beyond measuring and understanding these animal-driven dynamics, researchers are already using this work to judge how best to restore an endangered species, a process known as rewilding.

“We often want to rewild these exotic big animals because we have this fascination about them...but we often don’t really understand what’s going to happen,” says Diego Ellis Soto, a global change biologist and Yale doctoral student. Rewilding efforts that bolster animal populations could also disrupt nutrient cycling in the bodies of water they rely on to regulate their body temperature.

Ellis Soto has spent over a decade studying giant tortoises on Santa Cruz Island, a lush gem in Ecuador’s Galápagos archipelago. Along with other scientists contributing to the Galapagos Tortoise Movement Ecology Program, he tracks tortoise behavior with a goal of understanding how the animals change the chemistry of their environment.

**Figure:**
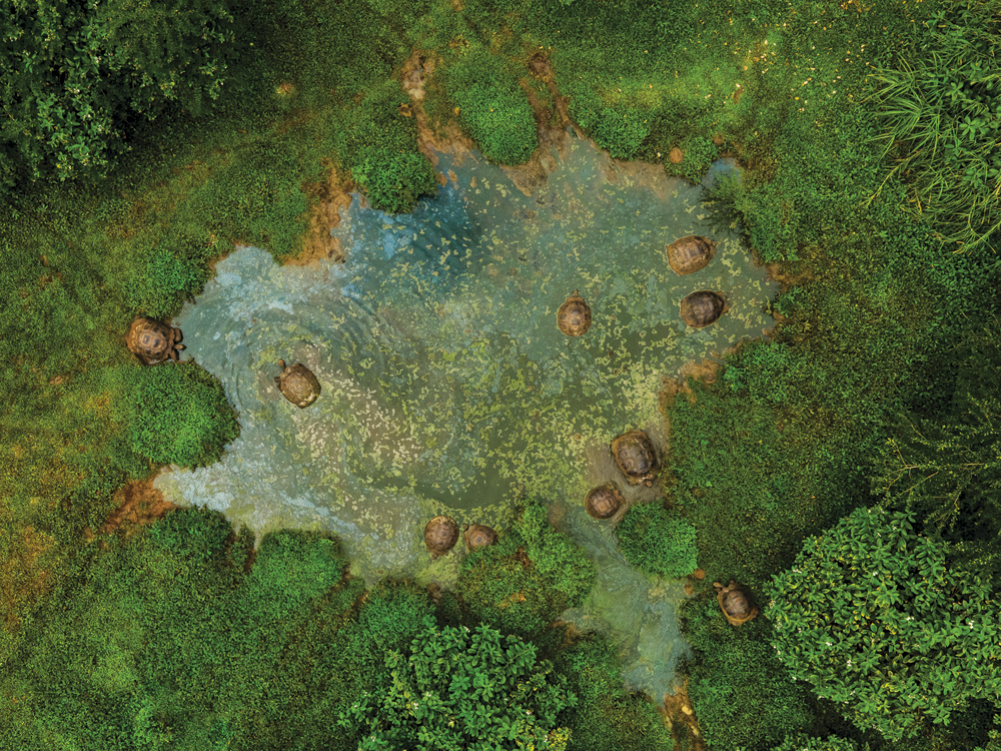
Cold-blooded Galápagos giant tortoises use freshwater ponds to regulate body temperature. Credit: Rashid Cruz/CDF.

“I like to think that giant tortoises are a mixture of a gardener and a mason because they disperse seeds, but they also move tons of nutrients around,” Ellis Soto says. The changes introduced by these unwitting chemical interlopers shift ecosystem productivity, spurring plant growth, bolstering insect populations, and more.

During the dry season, giant tortoises migrate to cool, wet highlands, congregating in freshwater ponds. Each giant tortoise slops a pound of mud from water to land as they clamber out, dragging the pond ecosystem’s waste and leaving “screaming high” levels of dissolved organic carbon and nutrients like ammonium and phosphate in their wake, according to Subalusky, whose laboratory helps analyze Ellis Soto’s samples. These chemicals likely come from decaying urine, feces, mud caked on tortoise legs, and agricultural runoff. Ellis Soto hopes that future studies will trace the nutrients from these organic byproducts to the pond’s mucky bottom in more detail to help scientists quantify the tortoises’ impact.

But new climate extremes now mean cold-blooded tortoises spend more time in freshwater ponds to regulate their body temperatures. This change in behavior increases the animals’ impact even more, as additional trips to thermoregulate mean more and more organic matter slopped into and out of the ponds.

## Choosing biodiversity alongside carbon capture

Zoogeochemistry adds a new dimension to climate impact research. Classically, scientists viewed animals as passive victims of climate change. Yet under certain conditions, they may amplify the effects of climate change, accelerating the pace of carbon emissions from their environments as they lengthen their stays in the ponds.

Subalusky says zoogeochemical studies could fuel nature-based efforts to mitigate climate change by investigating how animals might boost helpful carbon storage.

Scientists are already looking for ways to lock carbon away in plants—by strengthening kelp forests or mangrove stands, for example. But animals can store carbon too. The issue is they’re unpredictable; within a species, the same behaviors can yield carbon storage in one ecosystem and release in another, depending on a host of conditions, Subalusky says.

**Figure:**
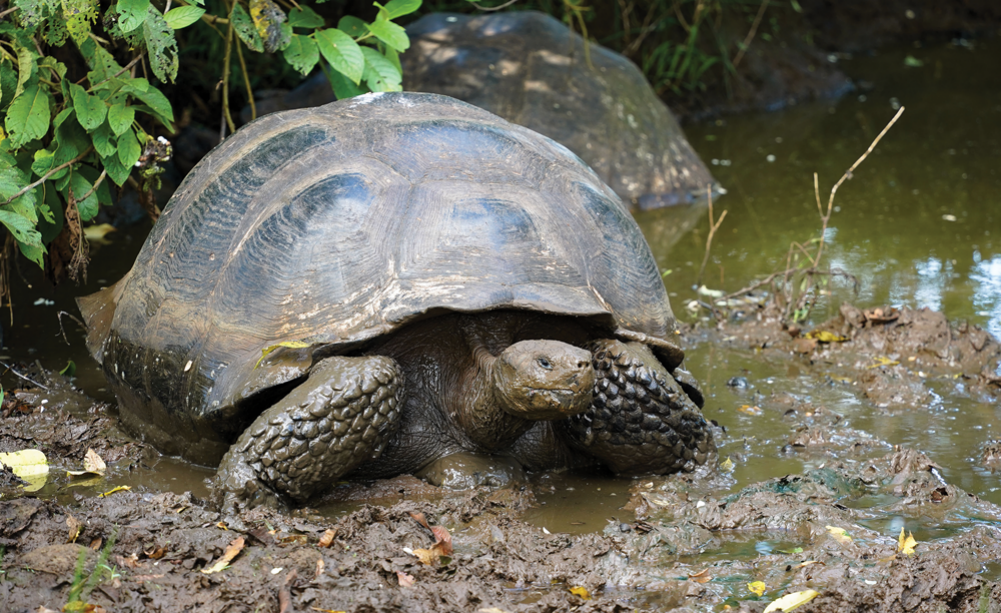
A giant tortoise hunkers down in the muck, which will soon be caked to its legs, making the animal a perfect vehicle for nutrient transport. Credit: Rashid Cruz/CDF.

Factors of interest include variations in ground cover abundance and permafrost thickness, as well as soil texture, moisture, and pH, according to Schmitz. Some types of soil, for example, are more conducive to storing carbon than others.

Likewise, varying conditions along the Mara River mean that not every pond will emit greenhouse gases even if hippo populations rebound. If the river’s water level rises, the waterway could flush decaying hippo waste from the ponds more frequently, tamping down the microbial activity that leads to methane emissions. Subalusky has witnessed climate factors like hotter, dryer weather altering animal impacts by shifting migratory patterns and feeding behaviors.

Amid these extreme changes, there’s no consensus among zoogeochemists on the best way to use or even communicate the data they’re collecting. Groffman believes the focus on methane emissions from animals’ activities could unintentionally add noise to the discussion on cutting greenhouse gas emissions. “Methane associated with hippos has nothing to do with climate change,” he says. “It distracts from the need to decarbonize the economy.”

Zoogeochemists tend to agree. Dutton, for example, says he is more worried about the cascading harms of hotter climates drying up rivers earlier in the season than he is about methane from hippos’ behaviors.

Schmitz wants these findings to be incorporated into conservation efforts that broaden their focus beyond single species. Such efforts “could be a win-win for actively mitigating both biodiversity loss as well as climate change,” he says.

There’s also trepidation that data linking animals to carbon emissions could be oversimplified and misapplied. “Biodiversity can increase the resilience of systems, even when maybe it’s not tipping the carbon balance quite the way you might want,” Subalusky says. So halting hippo conservation efforts, for example, because a particular herd’s activities led to increased methane emissions would be missing the bigger picture. The next steps are to hone predictions of the impact of rewilding animals, she says, not to prioritize species on the basis of potential carbon capture.

Ellis Soto hopes to share data on nutrient cycling with nongovernmental organizations and conservation groups as giant tortoise rewilding efforts continue. He says conservation must be balanced with a growing and potentially threatening tourist economy; the highland ponds are a popular destination despite the disruptions humans can cause to tortoise habitats.

Species- and ecosystem-specific data can “help us plan ecosystem restoration initiatives and also understand the risks of inaction or action,” he says. Unfortunately, giant tortoise data from the Galápagos can’t be applied to, say, tortoises in the Indian Ocean near Seychelles. The ecosystems are too different in countless ways, including climate, soil microbiota, and species makeup.

As zoogeochemists challenge the conservation paradigm, they’re also delivering new variables for policymakers and land managers to consider. Their research, if laborious, aims to spotlight and mend the threads that interweave animals and their habitats. “This work is exciting because it shows that the loss of a species is not just the loss of that species but also the loss of critical connections in ecosystems,” Ellis Soto says.

Closely considering animals as drivers of the same nutrient cycling that scientists have measured from the ocean floor to outer space won’t arrest climate change. But it may be a step toward quantifying all we stand to lose in its wake.

## Robin Donovan is a freelance contributor to

Chemical & Engineering News, *the independent news outlet of the American Chemical Society.*

